# Psychometric Properties of the Taiwanese Pressure Ulcer Management Self-Efficacy Scale in Nursing Practice

**DOI:** 10.3390/healthcare10101900

**Published:** 2022-09-28

**Authors:** Wen-Yi Chao, Yu-Lin Wu, Wen-Chun Liao

**Affiliations:** 1 Department of Public Health, China Medical University, Taichung 406040, Taiwan; 2 Department of Nursing, Nantou Hospital, Nantou 540234, Taiwan; 3 Post-Baccalaureate Program in Nursing, College of Nursing, Taipei Medical University, Taipei 11031, Taiwan; 4 School of Nursing, China Medical University, Taichung 406040, Taiwan; 5 Department of Nursing, China Medical University Hospital, Taichung 404332, Taiwan

**Keywords:** self-efficacy, pressure injury, nursing

## Abstract

Self-efficacy strongly predicts clinical performance and competence. In Taiwan, there is no reliable method for assessing self-efficacy in the management of pressure injury. This study aims to establish psychometric properties of the Pressure Ulcer Management Self-Efficacy Scale (PUM-SES) translated for Taiwan and determine the validity and reliability of the Taiwanese version of the PUM-SES. Materials and methods: The PUM-SES was translated for use in Taiwan using Brislin’s method. The translation’s content validity, concurrent validity, predictive validity, internal consistency, and test–retest reliability were evaluated. The Pressure Ulcer Management Self-Efficacy Scale, Taiwanese version (PUM-SES-T), the Attitude toward Pressure Injury Prevention Scale (APIPS) and the Practice toward Pressure Injury Prevention Scale (PPIPS) of preventing pressure injury, and the General Self-Efficacy Scale (GSES) were tested using Pearson’s correlation. A cross-sectional survey with 330 RNs in Taiwan was conducted. The PUM-SES-T was used to predict the PPIPS, and a predictive regression model was constructed considering nursing demographic variables. Results: Seven experts evaluated the PUM-SES-T with a CVI value of 0.995. An internal consistency, using Cronbach’s α, of 0.762 and a test–retest reliability of 0.997 were obtained. The PUM-SES-T was positively correlated with the GSES (*p* < 0.001). Multiple regression revealed that the PUM-SES-T predicted practice with a strong predictive validity (F = 8.077, *p* < 0.001), had an adjusted R^2^ of 0.455, but collinearity was insignificant. In this study, PUM-SES-T is a valid instrument for intervention-related educational programs to measure self-efficacy with good reliability and validity. It can be employed when intervening in related education strategies or promoting policies.

## 1. Introduction

Pressure injury (PI) is an essential indicator of caring quality, and its prevention is highly prioritized by nurses, health practitioners, and medical institutions worldwide. Nurses’ decision making during care is critical in preventing and managing PI [1,2]. The educational intervention that consisted of small-scale educational meetings, educational materials, and outreach visits improved the knowledge of PI prevention in hospital nurses [3]. Knowledge and attitude regarding PI were positively correlated with the clinical practice of PI prevention by nurses [4,5,6]. The prevention and management of PI is not a single technique or action but a series of processes that may be difficult to monitor. According to Bandura’s social cognition theory, self-efficacy refers to an individual’s perception of regulating their functioning, and self-efficacy affects every area of human endeavor [7]. Self-efficacy determines the initial behavior and performance when attempting to achieve a goal, i.e., “I can do it.” [7,8] Applying this theory to the field of nursing, self-efficacy involves events that shape a nurse’s clinical performance [9]. Nurses’ self-efficacy in managing PI can predict patient outcomes, thereby measuring nurses’ overall ability to deal with such conditions [10]. The General Self-Efficacy Scale (GSES), translated into multiple languages, was created to assess perceived self-efficacy. The GSES can predict the ability to cope with daily hassles and adapt to stressful life events [11]. According to Bandura, although this widely used scale is the gold standard, an individual’s perception of their behavioral abilities is specific to their confidence [12]. According to self-efficacy theory, the scales of perceived self-efficacy must be tailored to the particular domain of functioning that is the object of interest [12]. Caring behaviors in different health fields encounter unique situational obstacles and difficulties in the implementation process. Therefore, self-efficacy scales toward specific functioning are required [13], especially for PI management.

Most healthcare practitioners maintain a positive attitude toward PI prevention; however, converting the positive attitude into an actual strategy to prevent PI is challenging [14]. Several studies revealed that more positive attitudes toward PI prevention led to implementing more preventive behaviors [15]. The more PI prevention is valued, the greater the likelihood of conducting preventive practices [5].

Owing to the necessity of measuring nurses’ self-efficacy in preventing and managing PI, Dellafiore et al. [10] developed a short Pressure Ulcer Management Self-Efficacy Scale (PUM-SES) with 10 questions. Four dimensions are addressed by the questionnaire, including evaluation, planning, supervision, and decision-making. A multicenter cross-sectional approach study was conducted in northern Italy for psychometric evaluation. The four dimensions were positively correlated with good face and content validity, concurrent validity, construct validity, and reliability of internal consistency [10]. By implementing this scale, the four dimensions can be classified and analyzed to understand the extent to which the self-efficacy of nurses in the management of pressure injuries affects PI care as well as to formulate relevant care plans and education strategies [10].

Validated instruments can be translated into different languages with psychometric validation for optimal use. The Brislin translation process can be used to guide the cross-cultural translation [16]. According to Brislin’s, the adaptation and equivalence of the translated version can be confirmed through forward and backward translation and verification [17]. Psychometric analysis can be used to assess the reliability and validity of the translation process; the analysis must include at least two reliability test methods to examine the consistency and stability of measurement results, including internal consistency and retest reliability. The validation includes at least one content validity and one construct validity or criterion validity to detect whether the characteristic or concept can be measured. This study aimed to establish the psychometric properties of the Taiwanese version of PUM-SES (PUM-SES-T) for validity and reliability.

## 2. Materials and Methods

### 2.1. Design

**Figure 1:**
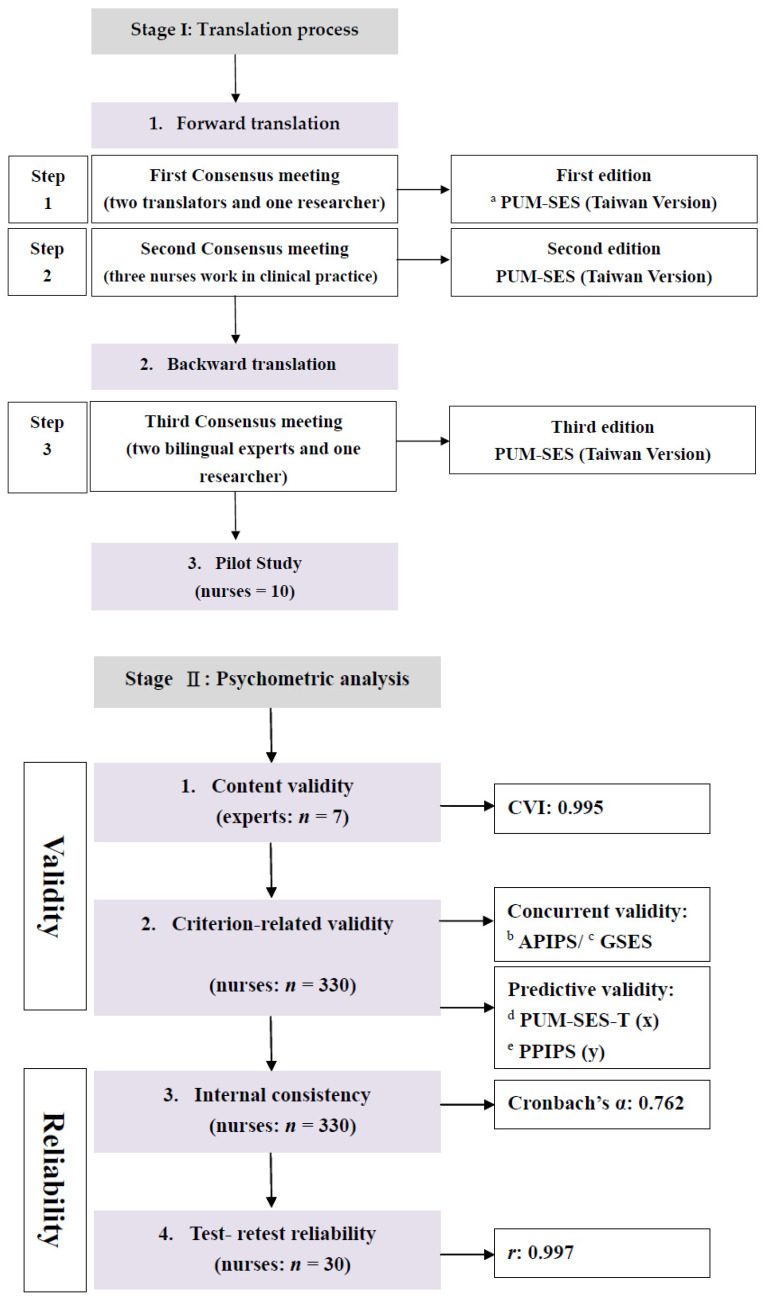
Step for translation and validation of the PUM-SES-T. ^a^ PUM-SES: Pressure Ulcer Management Self-Efficacy Scale. ^b^ APIPS: Attitude toward Pressure Injury Prevention Scale. ^c^ GSES: General Self-Efficacy Scale. ^d^ PUM-SES-T: Pressure Ulcer Management Self-Efficacy Scale Taiwanese version. ^e^ PPIPS: Practice toward Pressure Injury Prevention Scale.

This study was conducted in two stages. First was the translation of the PUM-SES. Second, a psychometric analysis was performed to evaluate validity and reliability (Figure 1). The Institutional Review Board approved this study (trial reference number: TPC109064). All participants were given verbal and written information about the study. Signed informed consent was obtained. Dr. Rosario Caruso approved the use of the PUM-SES and validated the PUM-SES-T.

### 2.2. Translation of the PUM-SES

The Brislin translation process was used to confirm the reliability and equivalence of the translated PUM-SES-T, using forward and backward translation and verification [16,17]. The translation was conducted in three steps. According to Brislin’s suggestion, iterative translations were used. First, two bilingual experts translated the source language of the scale into the target language, and the researcher checked the translation. The first consensus meeting confirmed the first version of the PUM-SES-T. Second, a second consensus meeting including three nurses engaged in clinical work was held to verify the version of the PUM-SES-T. The third step was a backward translation. Two bilingual experts who never witnessed the scale performed the back translation. If a question was not appropriately translated, the original text was compared with the translated version until the target and source language versions were culturally equivalent. For the third version of the PUM-SES-T, ambiguous meanings and semantics were removed. After completing the translation process, a pilot study was conducted in a regional teaching hospital with 10 nurses to confirm that the PUM-SES-T was easy to comprehend.

### 2.3. Psychometric Analysis

The study’s second stage included the psychometric analysis of the final version of the PUM-SES-T. The validity tests included content validity and criterion-related validity, and the reliability tests included internal consistency and test–retest reliability.

Participants for the psychometric evaluation study were recruited at a regional teaching hospital from April to May 2021. The inclusion criteria were nurses who (1) worked full-time or hourly salary in the research site and (2) agree to participate in the research and provide informed consent. Nursing students were excluded.

#### 2.3.1. Validity

Content validity, concurrent validity, and predictive validity were applied to evaluate the validity of the PUM-SES-T.

##### Content Validity

For content validity, seven experts, including the chief surgeon; a nursing professor; two international wound, ostomy, and continence nurses; two nursing administration supervisors; and a clinical nurse, evaluated the PUM-SES-T. Essential content evaluations included the degree of importance between the meaning of the questions and research topic, the wording of the question content, and the clarity of the semantic description. A content validity index (CVI) was used to calculate the content validity. The scoring was based on a 5-point Likert scale where 5 points indicated very important, clear, and certain, and 1 point indicated very unimportant, very unclear, and uncertain. If the rating was less than 3 points, content was discussed and revised based on experts’ suggestions. At this stage, expert revision of the PUM-SES-T was performed.

##### Concurrent Validity

Concurrent validity is a criterion-related validity, simultaneously measuring the relevance of the construct and objective indicators. The concurrent PUM-SES-T validity was assessed with the Attitude toward Pressure Injury Prevention Scale (APIPS) and the GSES and estimated using Pearson’s correlation.

##### Predictive Validity

The PUM-SES-T was employed to predict the practice of preventing and managing PI. Univariate and multivariate regression models were used to predict the PPIPS, and a predictive regression model was constructed considering nursing demographic variables.

#### 2.3.2. Reliability

Internal consistency and test–retest reliability were applied to evaluate the reliability of the PUM-SES-T.

##### Coefficient of Internal Consistency

The internal consistency reliability was verified by correlations among the 10 items of the PUM-SES-T scale and presented as Cronbach’s α. Values greater than 0.70 were considered acceptable [18].

##### Test–Retest Reliability

A small subset of the study participants was randomly selected to repeat the tests two weeks after the first test. Correlation coefficients were calculated from the scores of the two tests. Weighted kappa was used to estimate the test–retest reliability. The intraclass correlation coefficients values range from 0 to 1, with acceptable levels of 0.50–0.75, confident levels of 0.75–0.90, and perfect levels of >0.90 [19].

### 2.4. Measurement and Variables

#### 2.4.1. Participant Characteristics

Participant characteristics included individual demographic (gender, age, and academic qualification), work (professional category, career ladder, years of working experience, and working unit), and learning experience variables. The Taiwan Nurses Association defines the career ladder of clinical nurses’ roles and functions. Novice and beginner nurses with grading levels at N and N1 perform basic nursing, respectively; advanced beginners at N2 are competent in intensive nursing; qualified nurses at N3 are responsible for education and comprehensive nursing; and proficient nurses at N4 can conduct research as well as function as clinical nurse specialists.

A total of 330 nurses agreed to participate in this study and returned their questionnaires. A large proportion of them were aged 35–50 years (159, 48.2%) and more than half possessed a bachelor’s degree in nursing (57.3%). Almost all participants were clinical nurses (89.1%); 39.7% were at the N_2_ stage of the career ladder. With regard to working units, 41.2% of participants were medical and surgical ward nurses. Most participants had not obtained relevant PI certification licenses (98.2%) and had not participated in relevant PI training in the last year (85.8%). More than half of the participants had not read relevant books or articles (61.8%) or guidelines (53.0%) in the previous year. However, 66.7% had searched for relevant PI information using online resources (Table 1).

#### 2.4.2. Pressure Ulcer Management Self-Efficacy Scale Taiwanese Version (PUM-SES-T)

The PUM-SES-T was used to assess nurses’ self-efficacy in managing PI. Ten questions from the PUM-SES-T were classified into four dimensions, including evaluation, planning, supervision, and decision-making. A Likert 5-point scale, ranging from 1 (completely unable) to 5 (completely capable), with a total score of 10–50 was applied. In the original PUM-SES developed by Dellafiore et al. [10], the psychometric validation process was performed with data collection in two Italian hospitals with a convenient and consecutive sampling method in recruiting 182 nurses. Further, a random sample of 15 nurses was invited to rescale 20 days after the first assessment to determine the PUM-SES stability using a test–retest approach. The two measures of the test–retest was associated using the Pearson’s correlation (*r*), where a higher correlation indicated good stability. The PUM-SES scale revealed that four dimensions, including evaluation, planning, supervision, and decision-making of the consistency (Cronbach’s α) between 0.871 and 0.930, were positively correlated (*p* < 0.001), and the test–retest reliability was >0.60 (*p* < 0.001) [10].

#### 2.4.3. Attitude toward Pressure Injury Prevention Scale (APIPS)

The APIPS was used to assess the attitudes of nurses toward PI prevention. This scale consists of 11 items rated on a 5-point Likert scale, from 5 (strongly disagree) to 1 (strongly agree). Items 1, 6, 7, and 11 were reversely scored. Scores on this scale range from 11 (most negative attitudes) to 55 (most positive attitudes) [20]. The APIPS was commonly used in measuring nurses’ attitudes toward PI prevention [14,20], with good internal consistency reliability and Cronbach’s alpha of 0.91 [21].

#### 2.4.4. Practice toward Pressure Injury Prevention Scale

The PPIPS is a structured questionnaire [22,23,24] that was used to measure nurses’ practice toward PI prevention in this study. It consists of 20 items rated on a 5-point Likert scale, from 5 (always) to 1 (never). The total scores range from 20 to 100 [22]. Nurses answered correctly greater than or equal to 80% of the PPIPS test indicated good practice; less than 80% indicated poor practice [23].

#### 2.4.5. General Self-Efficacy Scale (GSES)

The GSES is a one-dimensional self-report instrument containing 10 questions. Multiple studies in 33 different languages support GSES’s validity and reliability. In samples from 23 nations, Cronbach’s alphas ranged from 0.76 to 0.90 with majority of alphas above 0.80 [25]. Questions such as “How much do you agree with this statement?” were asked for confidence in ten difficulties, which individuals may encounter, and scored on a 4-point Likert scale from 1 (incorrect) to 4 (always correct), with a total score of 10 to 40. Both the PUM-SES-T and the GSES have holistic and four-dimensional validity [10,11].

### 2.5. Statistical Analysis

This study assessed content validity, concurrent validity, predictive validity, internal consistency, and interrater reliability of the PUM-SES-T. Data were analyzed using SPSS 22.0 statistical package software (IBM Corp, Armonk, NY, USA). Descriptive statistics were used to describe the sample and the distributions of demographic variables; inferential statistics, such as Cronbach’s alpha and Pearson’s correlation, were used to measure the validity and reliability of the scales. Furthermore, to explore the relationship between the dependent variable (Practice toward Pressure Injury Prevention Scale) and the independent variable (Pressure Ulcer Management Self-Efficacy Scale Taiwanese version), correlation analysis and significance tests were conducted before the forecasting with multiple regression models.

## 3. Results

### 3.1. Translation Process of the PUM-SES

#### 3.1.1. Forward Translation

The PUM-SES-T was translated using the Brislin translation process. First, two bilingual experts translated the scale’s English language. The seventh item, concerning “healthcare assistants,” showed a difference in meaning between the two countries. In Taiwanese culture, “healthcare assistants” who assist in the implementation of PI prevention should be referred to as “nurse aides.” This process was confirmed in the first version of the PUM-SES-T. A consensus was achieved at the second meeting held by three clinical nurses. No unclear descriptions were detected, and thus, the second version of the PUM-SES-T was confirmed.

#### 3.1.2. Backward Translation

The third step involved backward translation by two native English-speaking professionals who were unfamiliar with the scale. During the backward translation process, the experts had some discrepancies on three items. Thus, the original and the translated versions were compared and make sure that the target and source language versions were culturally equivalent to determine the third version of the PUM-SES-T.

#### 3.1.3. Pilot Study

In pilot testing, we tested the third edition of PUM-SES (Taiwan version) with ten randomly selected (using a random number generator) clinical nurses from the teaching hospital to examine the semantic expressions of the scale. The revised version performed no major revision during this stage. 

### 3.2. Psychometric Evaluation of the PUM-SES-T

#### 3.2.1. Validity

##### Content Validity

The CVI value was 0.995. The expert consensus version of the PUM-SES-T was established.

##### Concurrent Validity

Correlation analysis was performed to examine the concurrent PUM-SES-T validity with the GSES and APIPS. The PUM-SES-T was significantly and positively correlated with the GSES (*r* = 0.615, *p* < 0.001). The APIPS was not correlated with PUM-SES-T (*r* = 0.027, *p* = 0.083) (Table 2).

##### Predictive Validity

PPIPS was the dependent variable, and the PUM-SES-T was the independent variable when using regression analysis to examine the predictive validity of the PUM-SES-T on PPIPS. Demographic variables were included in the regression model as control variables. Univariate regression analysis shows that the PUM-SES-T was a significant predictor of the PPIPS (Table 3, Model 1). The coefficient of the adjusted R^2^ of 0.455 indicated that the PUM-SES-T explained 45.5% of the total variation of PPIPS. In the multivariate regression using the demographic variables, differences between work units were substantial. The practice of PI prevention in nurses at general acute wards was better than in ICUs, with a regression coefficient of −0.292 (t = −2.448, *p* = 0.02) (Table 3, Model 2). The PUM-SES-T was still a significant predictor of the PPIPS after controlling for the work unit (Table 3, Model 2). The adjusted R^2^ of Model 2 was 0.453, indicating that the PUM-SES-T and work unit together explained 45.3% of the total variation of PPIPS.

#### 3.2.2. Reliability

##### Coefficient of Internal Consistency

The Cronbach’s coefficient of 0.762 in 330 nurses indicated a good internal consistent reliability for the PUM-SES-T.

##### Test–Retest Reliability

Thirty nurses were randomly sampled from the 330 nurses, and the same questionnaire was administered two weeks later. Test–retesting aims to measure the stability and consistency of the measurement across time for more rigorous research results. The reliability of the test–retest was 0.997, which indicates that the questionnaire is highly reliable.

## 4. Discussion

The cross-cultural translation of the PUM-SES-T in this study included three consensus meetings and semantic modification by following the Brislin translation process. The CVI of the PUM-SES-T was 0.995, which shows the content validity was compatible with the original PUM-SES. The Cronbach’s α score of 0.762 for the final version of the PUM-SES-T indicates good internal consistency. The significant positive correlation between the PUM-SES-T and the GSES also presented a satisfied concurrent validity. The PUM-SES-T has good reliability and validity and can be used for future clinical applications.

This study examines the predictive PUM-SES-T validity on PPIPS. The univariate regression analysis result shows the PUM-SES-T as a significant PPIPS predictor but is considered to give a weak dependent variable prediction. The results from the multiple regression model revealed that nurses from different work units represent varied practice levels. Nurses from medical and surgical wards were better than those from the ICU. The PUM-SES-T is a significant predictor, but most other predictors are not significant; thus, the R^2^ of the overall model is not high. For the regression model, the R^2^ of Model 2 was 0.503, and the adjusted R^2^ was 0.453, indicating the collinearity is insignificant in the model. Therefore, it needs to be expressed conservatively that the requirement of job training and clinical experience encountered in different work units may affect the practice result toward PI prevention and management.

According to Bandura’s social cognition theory, self-efficacy determines the initial behavior and performance when attempting to achieve a goal, i.e., “I can do it.” [7,8]. Applying this theory to nursing, self-efficacy involves events that shape a nurse’s clinical performance. The PUM-SES short 10-item scale distinguishes four dimensions and explores the self-efficacy of nurses in depth as well as assessing themselves in preventing and managing PIs. Although the outcome of PI prevention can quantify and track PI incidence in clinical situations, it is challenging to identify practical weaknesses within the care team; thus, self-efficacy can be used as a reference for assessing practice. A limitation of this study is the lack of correlation with actual clinical outcomes. Therefore, follow-up research can assess the incidence of PI that is compared with the PUM-SES.

The PUM-SES-T consists with four dimensions, nurses can use the scale to review their ability to assess patients at high risk of PI, formulate prevention and management, supervise care plans, make decisions based on practice, and develop care plans for PI patients. This can be a reference for self-advancement. In addition, Nursing researchers, administrators, and educators can apply this instrument to understand nurses’ self-efficacy across different dimensions of evaluation, planning, supervision, and decision making regarding PI. Results of the PUM-SES-T can become references for developing job training programs.

When applying PUM-SES-T as an instrument to check preventing and managing PI, it is recommended to distinguish four dimensions of self-efficacy and propose corresponding policy promotion. For example, it is recommended to promote education and training on PI’s physiological and pathological changes regarding assessment dimension deficiencies. Regarding planning dimension deficiencies, it is recommended that unit members be recruited to discuss prevention plans. Regarding supervision dimension deficiencies, it is recommended that the leader and members should be included together to review the implementation and supervision of the program regularly; regarding decision-making dimension deficiencies, it is recommended to share care experiences. The PUM-SES-T is an easy and validated tool to measure self-efficacy toward PI prevention and management.

## 5. Conclusions

The PUM-SES-T is a validated instrument with an appropriate psychometric evaluation of internal consistency, test–retest reliability, content validity, concurrent validity, and predictive validity. It is simple and convenient to assess self-efficacy toward preventing and managing PI in 3 to 5 min. Nursing administrators can use this instrument to identify nurses’ confidences and weaknesses in preventing and managing PI. The PUM-SES-T can be employed as a reliable instrument when intervening in related education strategies or promoting policies.

## Figures and Tables

**Table 1:** Characteristics of participants’ analysis (*N* = 330).

Variable		Frequency (n)	Percentage (%)
Gender			
	Male	21	6.4
	Female	309	93.6
Age (years)			
	<25	42	12.7
	25–34	111	33.6
	35–50	159	48.2
	>50	18	5.5
Academic degree			
	College	99	30.0
	Bachelor	189	57.3
	Master	42	12.7
Professional category			
	Primary nurse	294	89.1
	Nurse practitioner	26	7.9
	Nursing administrator	10	3.0
Career ladder ^a^			
	N	91	27.6
	N1	69	20.9
	N2	131	39.7
	N3	29	8.8
	N4	10	3.0
Working years			
	≤1	34	10.3
	2–5	64	19.4
	6–10	62	18.8
	11–15	60	18.2
	16–20	60	18.2
	≥21	50	15.2
Work unit			
	Medical and surgical wards	136	41.2
	Emergency room/ ICU	69	20.9
	Chronic unit ^b^	19	5.8
	Special unit ^c^	64	19.4
	Operation room	31	9.4
	Case manager/ Home care	11	3.3
Any certification			
	No	324	98.2
	Yes	6	1.8
PI ^d^ lecture attended ≤ 1y ago			
	No	283	85.8
	Yes	47	14.2
Read PI book or article ≤ 1y ago			
	No	204	61.8
	Yes	126	38.2
Sought information on PI via Internet			
	No	110	33.3
	Yes	220	66.7
Read PI guideline			
	No	175	53.0
	Yes	155	47.0

^a^ Career ladders. N, N_1_: Basic nursing skill. N_2_: Capable of critical care. N_3_: Nursing teaching ability. N_4_: Nursing research ability. ^b^ respiratory care ward, and nursing home. ^c^ special: hemodialysis room, outpatient department, psychiatry ward. ^d^ PI: pressure injury.

**Table 2:** Pearson correlation and mean score of PUM-SES-T, APIPS, and GSES.

	Scale Score		PUM-SES-T
Variables	Mean	SD	*r*
^a^ PUM-SES-T	27.382	7.6424	1.000
^b^ APIPS	35.718	5.4758	0.027
^c^ GSES	25.809	5.7530	0.615 **

^a^ PUM-SES-T: Pressure Ulcer Management Self-Efficacy Scale Taiwanese version. ^b^ APIPS: Attitude toward Pressure Injury Prevention Scale. ^c^ GSES: General Self-Efficacy Scale. *r* = Pearson correlation. SD = standard deviation. Score range: PUM-SES-T = 10–50; APIPS = 11–55; GSES = 10–40. ** *p* < 0.001.

**Table 3:** The predicting outcome of PUM-SES-T on PPIPS.

Variable	Description	Model 1	VIF	Model 2	VIF
		*β* (*t*)		*β* (*t*)	
Constant		0.000 (0.011)		0.176 (0.0476)	
PUM-SES-T	PPIPS	0.320 ** (6.915)	1.267	0.331 ** (6.300)	1.632
Age (Ref: < 25)	25–34			0.219 (1.169)	4.653
	35–50			0.322 (1.375)	8.126
	>50			0.179 (0.563)	3.112
Academic qualification (Ref: Bachelor)	College			−0.065 (−0.662)	1.192
	Master			0.175 (1.094)	1.703
Professional category (Ref: Primary nurse)	NP			0.144 (0.835)	1.284
	Administrator			0.284 (0.895)	1.775
Career ladders ^a^ (Ref: N_2_)	N			0.149 (1.050)	2.404
	N_1_			0.008 (0.062)	1.429
	N_3_			0.027 (0.163)	1.341
	N_4_			−0.324 (−1.115)	1.488
Years of working experience (Ref: 2–5)	≤1			0.205 (1.004)	2.309
	6–10			0.127 (0.872)	1.927
	11–15			−0.031 (−0.170)	2.916
	16–20			−0.094 (−0.434)	4.134
	≥21			−0.101 (−0.442)	3.986
Work unit (Ref: general ward)	ER/ ICU			−0.292 * (−2.448)	1.406
	chronic unit ^b^			−0.091 (−0.462)	1.252
	special unit ^c^			−0.099 (−0.770)	1.514
	operation room			−0.007 (−0.043)	1.328
	case manager/ home care			−0.334 (−1.296)	1.282
R square		0.460		0.503	
Adjusted R Square		0.455		0.453	

* *p* < 0.05, ** *p* < 0.01. ^a^ Career ladders. N, N_1_: Basic nursing skill. N_2_: Capable of critical care. N_3_: Nursing teaching ability. N_4_: Nursing research ability. ^b^ Respiratory care ward, and nursing home. ^c^ Special: hemodialysis room, outpatient department, psychiatry ward. Model 1: Univariate linear regression on practice. Model 2: Multivariate linear regression on practice by adjusting significant covariables from Model 1.

## Data Availability

Not applicable.
